# Cognitive orientation to daily occupational performance (CO-OP) approach as telehealth for a child with developmental coordination disorder: a case report

**DOI:** 10.3389/fresc.2023.1241981

**Published:** 2023-08-14

**Authors:** Hiroyasu Shiozu, Shigeki Kurasawa

**Affiliations:** ^1^ Department of Occupational Therapy, College of Life and Health Sciences, Chubu University, Kasugai, Japan; ^2^ Department of Occupational Therapy, School of Health Sciences, Fukushima Medical University, Fukushima, Japan

**Keywords:** CO-OP approach, telehealth, occupational therapy, coaching, developmental coordination disorder

## Abstract

**Aim:**

This study aimed to propose a possible interventional form of occupational therapy through a case study report of the applied Cognitive Orientation to daily Occupational Performance (CO-OP) approach as telehealth for a child with developmental coordination disorder (DCD).

**Methods:**

The intervention method was CO-OP-based tele-occupational therapy for a boy with DCD and his mother; 10 sessions were conducted using a video-conferencing system. This study used the Canadian Occupational Performance Measure (COPM) and the Performance Quality Rating Scale (PQRS) as assessment tools. The PQRS evaluated each occupational performance based on videos recorded during the online sessions and videos taken by the mother of the child.

**Results:**

The CO-OP approach improved COPM performance and satisfaction as well as PQRS scores in the following five goals: (1) handwriting, (2) column addition, (3) jumping rope, (4) playing on the bar, and (5) riding a bicycle.

**Conclusions:**

An online approach based on the CO-OP was realistic and effective, to some extent. Continuing to develop telehealth interventions in the future is recommended.

## 1. Introduction

Occupational therapy involves providing interventions to facilitate children in performing daily activities and other occupations such as going to school, playing, and engaging in leisure activities. Traditionally, occupational therapy services were delivered in a physical setting, involving the child and providing training to the parents and other family members (1). However, during the COVID-19 pandemic, many children with disabilities did not have access to occupational therapy services; resulting in occupational therapists choosing tele-occupational therapy as a service delivery model. According to the World Federation of Occupational Therapists, telehealth is “the use of information and communication technologies (ICT) to deliver health-related services when the provider and client are in different physical locations” (2). *Telehealth* refers to synchronous (i.e., real-time) interactions between the therapist and client (e.g., videoconference, remote monitoring, virtual interactions using applications, and gaming technologies) and/or asynchronous transmission of data (e.g., video, photos, and electronic mail) by the provider and/or the client (2). The changing healthcare environment and technological advancements have led to an increased demand for occupational therapy services provided through telehealth. However, a recent systematic review of telehealth interventions in occupational therapy for children with developmental disorders found a low level of evidence (3). Therefore, there is a need to further research and accumulate evidence on telehealth interventions in occupational therapy for children. Additionally, it is essential to develop more appropriate and effective telehealth interventions regarding occupational therapy for children through practical application.

Within this telehealth framework, this study adapted the Cognitive Orientation to daily Occupational Performance (CO-OP) intervention approach for a child with developmental coordination disorder (DCD). The CO-OP approach is “a client-centered, performance-based, problem-solving approach that enables skill acquisition through a process of strategy use and guided discovery” (4). For individuals with DCD, activity-oriented and participation-oriented approaches are recommended to improve general, fundamental, and specific motor skills (5). Among these, the CO-OP approach is beneficial as an evidence-based activity and participation approach. In addition, this approach is an intervention based on verbal interaction and coaching and is considered suitable for tele-occupational therapy. In fact, several reports of telerehabilitation are based on the CO-OP approach (6–8); however, these reports studied adolescents and adults, and the CO-OP approach in the tele-rehabilitation for children with DCD that has not yet been explored. Therefore, this study aimed to propose a possible future interventional form of occupational therapy through a practice report of the CO-OP approach as a telehealth tool.

## 2. Case description

The case participant was an eight-year-old boy diagnosed with DCD. He was a second-grade elementary school student receiving special-needs education services. He garnered a full-scale IQ score of 92 on the Wechsler Intelligence Scale for Children-Fourth edition (WISC-IV), with a Verbal Comprehension Index of 109, a Perceptual Reasoning Index of 82, a Working Memory Index of 100, and a Processing Speed Index of 81.

He had motor coordination problems even before preschool. He had issues with both gross and fine motor skills, and his mother was aware of this. He received a clinical diagnosis of DCD from a child psychiatrist at the age of four (no standardized physical test was used). Prior to entering elementary school, he underwent one year of physical therapy, but the services were terminated upon entering elementary school. After entering elementary school, he did not receive any further therapy services, yet he continued to experience difficulties with activities related to physical coordination. As a result, he and his mother independently practiced at home in an effort to improve his motor coordination issues, but the difficulties persisted, leading to the initiation of occupational therapy.

He could not receive occupational therapy services as a result of the COVID-19 pandemic. His mother agreed for him to receive the applied CO-OP approach as a telehealth intervention and included in this case study.

This study was conducted after informed consent was obtained from the parents. Approval was obtained from the Ethics Committee of the Kansai University of Welfare Sciences (Number: 20-06).

### 2.1. Outcome measures

#### 2.1.1. Canadian occupational performance measure (COPM)

The effectiveness of this study's intervention was assessed using the COPM on both the child and his mother. The COPM consists of five steps and three scores. First, the clients identify their occupational performance in the areas of self-care, productivity, and leisure and score their importance on a scale ranging from 1 (*not important*) to 10 (*extremely important*). Second, the clients prioritize up to five occupations to focus on during the intervention. Third, the clients rate their performance. Fourth, the clients rate their satisfaction with their performance of the identified occupational task, with higher ratings indicating better performance and greater satisfaction. Fifth, performance and satisfaction with performance are reassessed after an appropriate time interval, and changes in clients' perceptions of their occupational performance can be calculated (9). A clinically significant difference is a 2-point change in COPM performance and satisfaction ratings (9).

This assessment was conducted pre-intervention (session 1) and post-intervention (session 10) using a video conferencing system.

#### 2.1.2. Performance quality rating scale (PQRS)

This study used the Performance Quality Rating Scale (PQRS) as an observational measure of the performance quality of the client-selected, personally meaningful activities (4) that rated performance on a 10-point scale from 1 (*can't do the skill at all*) to 10 (*does the skill very well*). While several versions of the measure have been adopted since its inception, the present study used the Performance Quality Rating Scale—Operational Definitions (PQRS-OD), comprising task performance operational definitions for different points along the Likert scale (10). For children, the smallest real difference for the PQRS-OD varied from 0.69 to 0.89 (10), with a change of 1 in their score indicating a significant change. It was standardized through video recordings of the activities observed and rated. In this study, videos were recorded either by being recorded online or being taken by the mother. The mother's method consisted of filming the child's performance in the home program outside the session. In addition, the therapist asked the mother to film each skill goal three times and to film the child's entire body.

### 2.2. Intervention

The intervention adopted the CO-OP approach for our tele-occupational therapy. The CO-OP included four objectives: (i) skill acquisition, (ii) cognitive strategy use, (iii) generalization, and (iv) transfer of learning. Moreover, the CO-OP had seven key features to achieve these objectives: (i) client-chosen goals, (ii) dynamic performance analysis (DPA), (iii) cognitive strategy use, (iv) guided discovery, (v) enabling principles, (vi) parent significant other involvement, and (vii) intervention format. The elements of this intervention, corresponding to the seven characteristics, are listed in Table 1. In addition, this intervention was conducted by a certified CO-OP therapist under the International Cognitive Approaches Network (ICAN).

**Table 1:** Format of the CO-OP approach as telehealth.

	Seven key features of the CO-OP	Format of this approach
1)	Client-chosen goals	
	• Setting parameters • Daily activity log • PACS, COPM, PQRS	• COPM and PQRS were performed pre- and post-intervention. • COPM was impremented on the participant using a video conferencing system. • PQRS was impremented the way that videos recording of the activities are observed and rated in two ways: one was recorded online sessions and the other was taken by the mother.
2)	Dynamic performance analysis (DPA)	
	• Motivation • Task knowledge • Performance competence	The therapist analyzed DPA for performance problems through a video-conferencing system and the observation of videos taken by the mother of the child.
3)	Cognitive strategy use	
	• Global problem solving strategy • Domain specific strategy • Good strategy use	The therapist had a child use the Global Strategy (GS) (Goal-Plan-Do-Check) and encouraged them to discover domain-specific strategy (DSS). The GS was projected on the videoconference system and explained, and the discovered DSS were organized into slides.
4)	Guided discovery	
	• One thing at a time • Ask, don’t tell • Coach, don't adjust • Make it obvious	Guided discovery features were used. e.g.) When the therapist guides the discovery of playing on the bar strategies, the therapist observes the video with a client and asks the client the following questions: “Are the arms bent or extended?”, “Are the feet properly positioned when kicking the ground? Is it too close or too far?”, and “Is the body bent or extended when going around the bars?”. In addition, to facilitate DPA, the video was sometimes paused or viewed in slow motion. Eventually, the client discovered the “armadillo” strategy (bending his body when spinning on the bars) and used that strategy to achieve his goal.
5)	Enabling principles	
	• Make it fun • Promote learning • Work towards independence • Promote generalization and transfer	The enabling principle features were used. e.g.) When the therapist worked on school subject assignment goals such as handwriting and column addition, he did so at a task difficulty level that allowed him to “just right challenge” rather than conform to curricular standards. Also, when he discovered a good strategy, the therapist discussed with him how to make use of this strategy in school and made minor changes as needed. In addition, the child and his mother were asked to create a “strategy card” to carry with them so that they would not forget the strategy at school. Additionally, we made sure to check their practices at school in the next session.
6)	Parent significant other involvement	
	• Active participation to promote generalization and transfer	His mother participated in every session. The concept of CO-OP was explained to his mothers and an instructional video was provided so that they could review it at any time. The mother was actively involved in homework.
7)	Intervention format	
	• Program structure • Session structure • Mateials	This is a remote intervention using video conferencing system and by email. The session was conducted once a week for one hour.

CO-OP, cognitive orientation to daily occupational performance; COPM, Canadian occupational performance measure; PQRS, performance quality rating scale; PACS, pediatric activity card sort (Polatajko & Mandichi, 2004)

This intervention was conducted using a video-conferencing system (Zoom) and e-mail communication - 10 times, once a week, for one hour per session. In preparation for the intervention, the therapist coordinated the schedule with the mother of the child through e-mail correspondence. PDF data describing how to operate the video-conferencing system (including a list of required equipment, installation instruction for the application, and guidance on conference participation), a summary of the seven features of the CO-OP, and a daily log used to facilitate goal setting were sent via e-mail. Additionally, one day before each session, the therapist emailed the mother the schedule and session content, along with the ID of the video-conferencing system.

During the online sessions, both the child and his mother were paired together at all times. While this was partly because the video-conferencing system could only be operated by the mother, the primary purpose was to create an opportunity for the mother to actively participate in the session, collaborate in the DPA process with the therapist and child, and encourage the child to discover strategies by prompting the mother. Visual support in the form of slides and verbal conversation were used by the therapist to facilitate interaction on the video-conferencing system. The slides included basic content such as; (i) the session schedule, (ii) the illustrated global strategy (Goal-Plan-Do-Chech), (iii) a list of goals selected by the client and their importance, performance and satisfaction scores, (iii) a list of domain specific strategies discovered by the client, (iv) a video of the client's performance scenes embedded in the slides used to collaboratively implement the DPA, (v) a free space slide for client comments in summary etc., and (vi) a list of activities to be implemented for homework. After each session, a PDF version of these slides was emailed to the mother for information sharing purposes.

For tasks that could not be performed during the session (e.g., gross motor tasks), goal setting, DPA, strategy planning, assessment of the quality of performance, and effectiveness of the strategies were conducted during the online session. However, the actual practice using the strategies was assigned as homework. To facilitate this, mothers were requested to video record the child's homework practice and report back to the therapist through email before the next session. This allowed the therapist to provide feedback and make any necessary adjustments to the intervention plan.

## 3. Findings

### 3.1. COPM: perceived performance and satisfaction

By making use of the COPM, the therapist identified five goals for the child and his mother: (i) handwriting, (ii) column addition, (iii) jumping rope, (iv) playing on the bar, and (v) riding a bicycle. For these goals, he reported a COPM-perceived performance and satisfaction changes that were at least two points higher for all tasks post-intervention (Table 2).

**Table 2:** Results of COPM and PQRS.

COPM						PQRS	
Occupations/Goals	Importance	Perfomrnace		Satisfaction		Pre	Post
		Pre	Post	Pre	Post		
Handwriting	8	4	9	3	10	4	10
Column addition	8	3	9	1	9	4	10
Jumping rope	10	5	10	5	10	2	10
Playing on the bar	10	1	8	1	8	6	8
Riding a bicycle	10	5	10	5	10	3	10

COPM, Canadian Occupational Performance Measure; PQRS, Performance Quality Rating Scale.

### 3.2. PQRS: observed performance

Of the five goals, the PQRS for handwriting and column addition were scored on videos recorded during online sessions. In addition, for jumping rope, playing on the bar, and riding a bicycle, the PQRS was scored during a video recorded by the child's mother. Note that for the purpose of scoring the PQRS-OD, the therapist created the definitions for scoring at the time of the initial observation of each activity. This approach ensured consistency and accuracy in the scoring process.

The PQRS results showed an improvement in the quality of execution of all goals: from 4 to 10 points for handwriting, 4–10 points for column addition, 2–10 points for jumping rope, 6–8 points for playing on the bar, and 3–10 points for riding a bicycle (Table 2).

### 3.3. Intervention implementation

**Figure 1:**
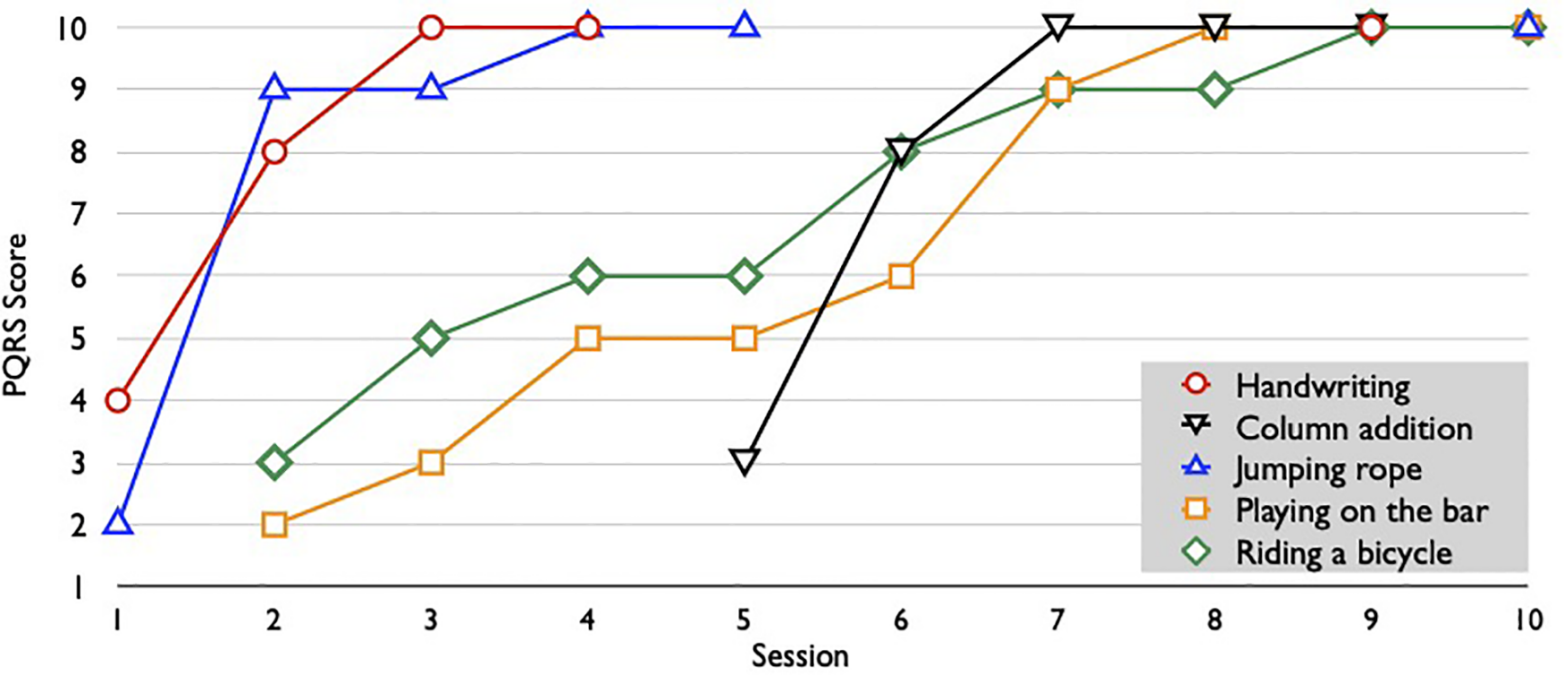
Tasks and PQRS scores per session. This graph shows the tasks addressed in each session and the progress of the Performance Quality Rating Scale (PQRS) scores. Each session had 2–4 tasks. However, jumping rope, playing no the bar, and riding a bicycle were using video to analyze performances, score, and discuss strategies for home programs.

Figure 1 exhibits the progress of the target tasks and the PQRS scores given during the intervention. Of the five goals, handwriting and column addition were performed within the online session. The other goals of jumping rope, playing on the bar, and riding a bicycle were on DPA and guided strategy discovery mode by the client and the occupational therapist using video observation during the online session. Practicing with the implementation of the strategies was a home program.

All goals were competencies to be implemented in schools and communities; thus, skill generalization and transfer were discussed in each session. As an example of generalization, a strategy discovered in column addition was “calculation using rap (rhythm and voice).” However, this strategy could not be used in class because there is a culture in this classroom that students are not allowed to speak up during exams; thus, after discussing alternative strategies, the therapist moved to a strategy called “rap calculation in the mind (using self-talk).” As a result, all these goals can be transformed into successful and satisfactory performances in schools and communities. For an example of transfer, the therapist found that the strategy of calculation using rap could be used for “handwriting kanji characters,” and he transferred this strategy.

## 4. Discussion

This case report studied the CO-OP approach for telehealth for boys with DCD. The current results exhibited that even when the intervention format was telehealth, implementing the CO-OP approach improved the child's perceptions of performance and satisfaction as well as the quality of performance.

All goals for the child were achieved, as assessed through COPM and PQRS. This result can be attributed to the features of the CO-OP approach. Its main feature is coaching. In telehealth, occupational therapists and clients are in different physical spaces and cannot physically interact. Therefore, intervention through verbal interaction is important, and occupational therapists need to make use of coaching skills. Coaching has been identified as one of the key skills of occupational therapists in the Canadian Model of Client-Centered Enablement (CMCE) (11), and evidence of good results from coaching has been demonstrated in recent years (12, 13). Our findings exhibited that coaching interventions that are effective in face-to-face practice are also applicable to telehealth.

However, limitations specific to telehealth owing to task constraints were identified. The online session could not target tasks involving gross motor activities (such as the jumping rope, playing on the bar, riding a bicycle) as a result of the viewing limitations of the camera angle and the limited physical environment. However, our practice addressed gross motor activities by implementing strategies in consultation with the child in an online session while reviewing recorded videos of the child performing activities during their homework sessions (Plan), using the strategies found in the practice as a home program (Do), and then confirming (Check) and providing feedback using the slides exhibiting the Goal-Plan-Do-Check in the subsequent online sessions. Additionally, mothers may access consultation interventions through various means such as phone calls, video conferencing, and e-mail, among others (4). This study suggests the possibility of overcoming the limitations unique to telehealth through the creative implementation of possible solutions. In this study, we placed particular importance on the active participation of parents. In telehealth interventions, the physical separation between the therapist and the child-mother facilitates their collaboration. Additionally, the CO-OP has demonstrated its efficacy in supporting children's occupational performance without the need for additional coaching approaches to parents (14). We therefore identified a synergy between the CO-OP and telehealth intervention. Although there were no other problems in this practice, the equipment used and the internet environment naturally influenced the implementation of telehealth. As a result, telehealth presents challenges for both service providers and clients in terms of internet infrastructure.

### 4.1. Limitations

This study demonstrated that implementing CO-OP through telehealth improved the occupational performance of a child with DCD. However, this study included the results of a single case and therefore needs to be validated with a quantitative research design in the future. In addition, the authors suggest that the adaptation of CO-OP as a telehealth strategy for the subject population should be verified to ensure that the practice is valid and reliable. There have been several reports of occupational therapy telehealth interventions for autism spectrum disorders (15, 16). However, there remains a scarcity of studies on children with DCD, similar to this study, and further quantitative research on telehealth interventions for children with DCD need to be conducted. Moreover, it is desirable to conduct intervention studies using CO-OP, which has demonstrated efficacy in face-to-face interventions (5), as employed in this study.

## 5. Conclusions

In this case study, the child's occupational performance improved with CO-OP through telehealth. These improvements were made possible by CO-OP's unique features, which are primarily based on a verbal interaction approach to coaching. In addition, the unique telehealth issue of task constraints was overcome through a guided strategy discovery online and the implementation of a home program. Further research and development of CO-OP for telehealth will benefit more children and families.

## Data Availability

The original contributions presented in the study are included in the article/supplementary material, further inquiries can be directed to the corresponding author.
